# Pediatric MASLD in China: epidemiology, screening, diagnosis, and management

**DOI:** 10.1016/j.lanwpc.2025.101717

**Published:** 2025-10-18

**Authors:** Xiaoguo Li, Xiao-Dong Zhou, Jie Wu, Zhenhua Zhao, Feng Xie, Yiling Li, Wenhui Li, Xiaosong Yan, Sumin Sui, Liting Zhang, Ming-Hua Zheng, Yuemin Nan, Xiaolong Qi

**Affiliations:** a Liver Disease Center of Integrated Traditional Chinese and Western Medicine, Department of Radiology, Zhongda Hospital, Medical School, Southeast University, Nurturing Center of Jiangsu Province for State Laboratory of AI Imaging & Interventional Radiology (Southeast University), Nanjing, China; b Basic Medicine Research and Innovation Center of Ministry of Education, Zhongda Hospital, Southeast University; State Key Laboratory of Digital Medical Engineering, Nanjing, China; c Department of Hepatology, First Hospital of Lanzhou University, Lanzhou, China; d MAFLD Research Center, Department of Hepatology, The First Affiliated Hospital of Wenzhou Medical University, Wenzhou, China; e Department of Gastroenterology, Beijing Children's Hospital, Capital Medical University, National Center for Children's Health, Beijing, China; f Department of Radiology, Shaoxing People's Hospital (Shaoxing Hospital of Zhejiang University), Shaoxing, China; g Functional Inspection Center, Lanzhou Maternal and Child Health Care Hospital, Lanzhou, China; h Gastroenterology Department, The First Affiliated Hospital of China Medical University, Shenyang, China; i Department of Hospital Administration, Yancheng Maternal and Child Health Care Hospital Affiliated to Yangzhou University, Yancheng, China; j Department of Surgery, Third People's Hospital of Tibet, Lhasa, China; k Department of Pediatric, The People's Hospital of Bozhou, Bozhou, China; l Department of Traditional and Western Medical Hepatology, Hebei Medical University Third Hospital, Shijiazhuang, China

**Keywords:** Pediatric metabolic dysfunction-associated steatotic liver disease, Childhood obesity, liver steatosis, Non-invasive screening, Epidemiology in China, Lifestyle intervention

## Abstract

Metabolic dysfunction-associated steatotic liver disease (MASLD) is an emerging global epidemic, with a rapidly increasing burden among children and adolescents in China. This alarming trend is primarily driven by profound lifestyle shifts. Unlike earlier perceptions of pediatric MASLD as relatively benign, pediatric-onset MASLD is now recognized as a progressive condition associated with liver fibrosis, cirrhosis, cardiometabolic comorbidities, and elevated risks of hepatocellular carcinoma and early mortality. In China, this escalating burden is compounded by regional disparities, diagnostic challenges, and limited access to pediatric-specific screening and management tools. Yet early-life detection and intervention present a critical window to mitigate long-term liver-related and systemic health consequences. This review synthesizes the latest epidemiological data, screening strategies, diagnostic innovations, and therapeutic approaches for pediatric MASLD in the Chinese context. By integrating emerging evidence with national public health priorities, we aim to inform actionable policies and tailored intervention strategies to confront this growing epidemic.

## Introduction

Metabolic dysfunction-associated steatotic liver disease (MASLD), formerly known as non-alcoholic fatty liver disease (NAFLD), has become the most common chronic liver disease in children worldwide, closely paralleling the global surge in pediatric obesity and metabolic dysfunction over the past five decades.^1^, ^2^, ^3^, ^4^, ^5^, ^6^ MASLD affects an estimated 13% of the general pediatric population aged 18 and under, yet it often goes undiagnosed.^7^^,^^8^ Nearly two-thirds of affected individuals remain untreated, placing them at risk of disease progression into adulthood.^7^^,^^8^ This silent course underscores MASLD as a significant contributor to the global burden of chronic liver disease in the pediatric population and a growing public health concern.^9^

In 2023, a pivotal shift occurred with the international consensus redefining NAFLD as MASLD, emphasizing hepatic steatosis with at least one of five cardiometabolic risk factors.^3^ This redefinition better reflects the disease's metabolic underpinnings and has significant implications for early detection and intervention, particularly in pediatric populations. Pediatric MASLD demonstrates distinct histopathological features compared to adults. Children more frequently exhibit severe steatosis with periportal inflammation and fibrosis, often without the ballooning degeneration typically seen in adult steatohepatitis.^3^ These differences highlight the need for age-specific diagnostic criteria and treatment approaches. The spectrum of steatotic liver disease (SLD) in children ranges from simple steatosis to more advanced forms involving inflammation, fibrosis, and cirrhosis.^10^ Notably, in children with biopsy-confirmed MASLD followed for an average follow-up of 1.6 years, disease progression was common, with 18% developing new-onset steatohepatitis and 23% experiencing worsening fibrosis.^11^ In a long-term retrospective cohort study involving 1096 children aged 2–18 years with MASLD and a mean follow-up of 8.5 years, all-cause mortality rate of 398 per 100,000 person-years, with 3.4% of children dying, nearly half from liver-related causes.^12^ Even with the standard of care, an estimated one-fifth of pediatric MASLD patients may experience disease progression.^13^ Moreover, youth-onset MASLD conferred a 5-fold higher mortality risk with simple steatosis and 11.5-fold with metabolic dysfunction-associated steatohepatitis (MASH) versus controls.^3^

Liver fibrosis is a key factor contributing to poor outcomes in patients with MASLD.^14^ Histopathologic studies show that fibrosis can range across all stages in affected children. Among children with biopsy-confirmed MASLD who are overweight or have obesity, up to 77% exhibit some degree of fibrosis, with 27% showing significant fibrosis (≥F2).^15^ Another study reports that nearly 10% of these children already have advanced fibrosis.^16^ Longitudinal studies indicate that approximately 6% of children with MASLD progress to advanced fibrosis within a decade, with some requiring liver transplantation in early adulthood, a trajectory notably earlier than seen in adult-onset disease.^17^ MASLD is closely linked not only to liver-specific complications but also to a wide range of cardiometabolic comorbidities.^18^ In children with MASLD, 23.4% have prediabetes, and 6.5% develop type 2 diabetes, with girls showing significantly higher risks of both conditions compared to boys.^19^^,^^20^ Elevated blood pressure is also common, affecting up to one-third of children with MASLD, with 7.2% requiring antihypertensive treatment.^21^

These global concerns are highly relevant to China, which bears the highest absolute global burden of liver disease, historically dominated by chronic hepatitis B.^22^ However, in recent decades, the country has undergone a rapid epidemiological transition driven by changes in diet, sedentary lifestyle, and rising childhood obesity.^1^ These changes have led to a sharp increase in MASLD among Chinese children and adolescents across both urban and rural populations. This escalating burden is compounded by regional disparities, diagnostic challenges, and limited access to pediatric-specific screening and management tools. The current epidemiological surveillance system for MASLD in Chinese children is incomplete.^7^ There is a lack of nationwide cohort studies and national guidelines for pediatric MASLD screening.^7^ Additionally, screening is restricted by region and economy, with rural areas lacking professional medical resources. This has contributed to low screening coverage and a rising burden of MASLD among Chinese children and adolescents. Therefore, addressing pediatric MASLD in China is critical, not only to reduce future liver-related complications, but also to curb the long-term burden of noncommunicable diseases on public health systems.

**Fig. 1:**
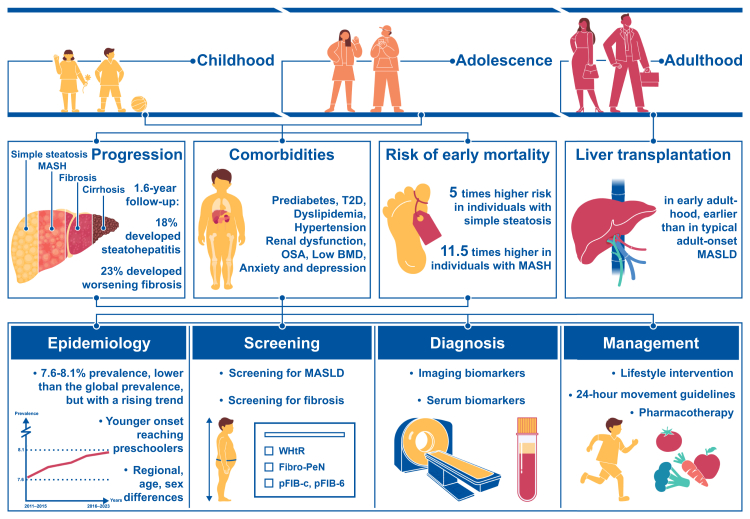
Progression and Current Landscape of Pediatric MASLD in China: From Early Disease Development to Advances in Epidemiology, Diagnosis, and Management. MASLD, metabolic dysfunction-associated steatotic liver disease; MASH, metabolic dysfunction-associated steatohepatitis; T2D, type 2 diabetes; OSA, obstructive sleep apnea; BMD, bone mineral density; WHtR, the waist-to-height ratio; Fibro-PeN, fibrosis in pediatric non-alcoholic fatty liver disease.

While several review articles have addressed pediatric MASLD,^23^^,^^24^ there remains a notable gap in comprehensive, population-specific summaries focused on Chinese children. Previous reviews have largely addressed broader epidemiological trends, pathophysiology, and management strategies in general pediatric populations, but they have not thoroughly considered the unique sociocultural, environmental, and healthcare system factors influencing pediatric MASLD in China. This review aims to fill that gap by providing a tailored analysis of the epidemiology, screening, diagnosis, and management for Chinese pediatric patients, contextualizing the evidence within China's distinct healthcare landscape and public health policies. By emphasizing China's unique demographic and clinical context, we identify key opportunities for early intervention and long-term disease prevention, with implications for global MASLD strategies (Fig. 1).

## Search strategy and selection criteria

A comprehensive literature review was performed on PubMed, Web of Science, CNKI, WanFang, VIP, Embase, and Scopus databases to identify pertinent full-text articles related to pediatric MASLD in China over the past 15 years. Additionally, government public documents related to childhood obesity and MASLD were also consulted. Given that the MASLD terminology was only established in 2023,^3^ current literature on MASLD remains limited; therefore, the data presented below are based on historical nomenclature and diagnostic criteria for NAFLD, MAFLD, MASLD, and SLD. The search strategy employed a broad set of keywords and their synonyms, including non-alcoholic fatty liver disease∗, non-alcoholic steatohepatitis∗, NAFLD∗, NASH∗, metabolic dysfunction-associated fatty liver disease∗, metabolic-associated fatty liver disease∗, MAFLD∗, metabolic dysfunction-associated steatotic liver disease∗, metabolic dysfunction-associated steatohepatitis∗, MASLD∗, MASH∗, child∗, adolescent∗, school-aged∗, pediatric∗, China∗, Chinese∗, mainland China∗, East Asian∗ in different combinations and with various synonyms. Each section of the search was tailored with specific keywords like epidemiology∗, prevalence∗, incidence∗, risk factors∗, screening∗, early diagnosis∗, ultrasonography∗, elasticity imaging techniques∗, early detection∗, controlled attenuation parameter∗, CAP∗, diagnostic techniques and procedures∗, biopsy∗, magnetic resonance imaging∗, diagnostic accuracy∗, life style∗, diet∗, vitamin E∗, drug therapy∗, lifestyle intervention∗, exercise∗, management∗, health policy∗ and guideline∗ to refine the results. The age range covered in the reviewed literature was limited to individuals up to 18 years old.

## Epidemiology

Pediatric MASLD in China has shown a steady increase over the past decade, reflecting national shifts in dietary habits, reduced physical activity, and rising childhood obesity.^1^ This trend is supported by a large population-based meta-analysis involving 17.4 million individuals reported a prevalence of 7.6% in children under 18 during 2011–2015, which rose to 8.1% between 2016 and 2023, signaling a gradual upward trend.^25^ Although this figure is lower than the global average of 13% and the American prevalence of 10–16%, it closely mirrors the Asian regional average of 7.0%, and continues to rise in tandem with China's epidemiological transition.^3^^,^^26^^,^^27^

Notably, the prevalence of MASLD is significantly higher in overweight and obese children compared to the general pediatric population. Meta-analyses have reported that the prevalence of MASLD in overweight/obese children in China was 40.4%–45.5%.^28^, ^29^, ^30^ Moreover, pediatric MASLD is detectable even in preschool-aged children, with one birth cohort study in Shanghai reporting a prevalence of 0.5% at age 5, increasing to 3.4% by age 8.^31^ These findings suggest that disease onset may occur much earlier than previously assumed, underscoring the need for early-life preventive strategies.

Substantial regional disparities have emerged across China, likely driven by geographic, dietary, and socioeconomic heterogeneity. In southern and eastern regions such as Wenzhou and the Yangtze River Delta, prevalence estimates among school-aged children generally fall between 4.4% and 5%. For example, a cross-sectional study in Wenzhou involving 7759 children aged 5–13 years reported a prevalence of 4.4%^32^ A multi-regional study across the Yangtze River Delta involving 7229 children aged 7–18 years reported an overall prevalence of 5.0%^33^ In contrast, northern cities such as Beijing, Xi'an, and Shenyang show substantially higher prevalence rates, ranging from 7.0% to 23.8%. In Beijing, among 1350 children aged 6–8 years, the prevalence reached 7.0%.^34^ In Xi'an, a study of adolescents aged 15–22 years found a prevalence of 8.1%.^35^ In Shenyang, among school-aged children and adolescents aged 7–18 years, the overall prevalence was as high as 23.8%, with similar rates across elementary (23.0%), middle (25.1%), and high school (23.4%) students.^36^ This result is consistent with the findings from a meta-analysis, which showed that the prevalence of MASLD in the pediatric population is higher in northern regions (7.1%) than in southern regions (6.0%).^30^ Two meta-analyses show that the prevalence of MASLD in obese children is significantly higher in southern and eastern cities compared to northern cities. One study, which included 16,481 overweight/obese children and adolescents, found significant regional variation in MASLD prevalence across: East China (50.7%), South China (46.7%), Southwest China (38.2%), North China (34.2%), and Northwest China (45.6%)^28^ Another study involving 7649 obese children, reported a higher prevalence in southern regions (48.4%) compared to northern regions (24%).^30^

Overall, the prevalence of MASLD among the general pediatric population in northern cities was higher than that in southern and eastern cities. However, in the high-risk group of obese children, the regional distribution of MASLD prevalence in southern cities was higher than that in northern cities (Supplementary Figure 1). This suggests that risk factors may vary across different regions: northern regions may have environmental factor that makes the general pediatric population more susceptible, while the southern regions may present a more severe metabolic risk for obese children. These regional disparities are likely influenced by differences in geography, dietary patterns, and socioeconomic factors.^37^, ^38^, ^39^

Sex-based differences are another consistent feature across epidemiological studies. Data from multiple regions indicate that boys appear to be at significantly higher risk of developing MASLD compared to girls, as seen in Wenzhou (5.6% vs. 1.9%),^32^ the Yangtze River Delta (7.5% vs. 2.5%),^33^ and northern cities including Beijing (10.4% vs. 3.7%).^34^ This trend is particularly pronounced among children with obesity, with MASLD prevalence reaching 49.2% in males compared to 33.1% in females.^28^ These differences may be partially explained by sex-specific variations in visceral adiposity, insulin resistance, hormonal regulation, pubertal timing, and lifestyle behaviors such as diet and physical activity.^19^^,^^20^ An important physiological distinction lies in fat distribution: males tend to accumulate more abdominal and visceral fats (Android fat), increasing MASLD risk, while females are more likely to store white adipose tissue in subcutaneous and femoral regions (Gynoid fat).^40^ Hormonal influences further contribute to this disparity. In boys, MASLD prevalence positively correlated with estradiol (p = 0.011) and negatively with testosterone (p < 0.001). In contrast, girls show an inverse relationship between MASLD and estradiol (p = 0.031). Additional associations include lower luteinizing hormone levels in boys and lower prolactin levels in girls with MASLD.^41^ The consistent male predominance across diverse settings suggests a biological and behavioral basis for differential susceptibility and highlights the importance of tailoring health education and intervention strategies accordingly.

In summary, while the overall prevalence of pediatric MASLD in China remains lower than that of many Western countries, national data indicate a steady increase over time, with evidence of early onset even in preschool children. The disease demonstrates marked geographic variation: specifically, MASLD prevalence among children in general is higher in northern cities than in southern and eastern regions. Conversely, among obese children, southern cities show a higher prevalence compared to northern areas. Additionally, sex-based differences are observed, with boys being at significantly higher risk than girls. These distinct patterns emphasize the need for public health strategies tailored to specific regions and genders, as well as the implementation of early screening programs and longitudinal research using standardized diagnostic criteria to fully elucidate the evolving epidemiological landscape of pediatric MASLD in China.

## Screening and early detection

Early identification of pediatric MASLD is critical for mitigating disease progression and preventing long-term hepatic and cardiometabolic complications. Recognizing this, the American Association for the Study of Liver Diseases (AASLD) recently issued a practice statement recommending targeted screening in high-risk children.^3^ Specifically, screening is advised for children aged ≥10 years who are obese, overweight with additional cardiometabolic risk factors, or with a family history of MASLD.^3^ Due to limited availability and cost of imaging modalities such as ultrasound or elastography in many settings, serum alanine aminotransferase (ALT) remains the primary recommended screening tool.^42^ However, ALT alone is imperfect in both sensitivity and specificity, particularly for early-stage steatosis or fibrosis.

This has prompted growing interest in anthropometric indices as alternative or adjunctive tools for early screening. In a large-scale cross-sectional study involving 16,914 children and adolescents aged 7–17 years from six cities in China, the waist-to-height ratio (WHtR) demonstrated strong diagnostic utility for identifying metabolic syndrome, with sensitivity and specificity of 0.86 and 0.83 in boys, and 0.87 and 0.81 in girls, respectively.^43^ The study recommends using WHtR cut-off values of 0.47 for boys and 0.45 for girls as key thresholds for early detection of childhood obesity and metabolic syndrome among Chinese children and adolescents. The findings suggest that these thresholds consistently offer strong sensitivity and specificity for diagnosing metabolic syndrome across different age groups.^43^ Further supporting its clinical value, a school-based study of 1018 children aged 6–14 years found that WHtR was the most effective non-invasive predictor for MASLD.^7^ Based on training and validation cohorts, a WHtR threshold of ≥0.48 was proposed as an optimal cut-off, especially for regions where the prevalence of childhood obesity exceeds 12%, and a WHtR ≥0.48 in regions where obesity prevalence ≥12.0%. For areas where the obesity data are unavailable, screening should target children with WHtR ≥0.48 and lipid accumulation product ≥668.22 cm × mg/dL.^7^ These findings highlight WHtR as an accessible and scalable metric for community-based screening in pediatric populations, particularly in resource-limited settings. However, the cost-effectiveness and performance of WHtR as a screening tool should be further validated in diverse populations and ethnic groups.

Beyond the detection of steatosis, screening for liver fibrosis, the key prognostic determinant in MASLD, is essential for risk stratification and guiding management decisions. Several non-invasive prediction models have recently been developed using routine clinical and laboratory data. Wang et al. proposed the fibrosis in pediatric non-alcoholic fatty liver disease (Fibro-PeN) model, which incorporates 14 widely available clinical parameters in a cohort of 1055 children with SLD.^44^ The model demonstrated good predictive performance for moderate-to-severe fibrosis, with an area under the curve (AUC) of 0.79 (95% CI: 0.77–0.81), sensitivity of 72%, and specificity of 76%. While external validation is needed, this model shows potential for use in both general practice and specialized pediatric settings. In addition, two fibrosis-specific scoring tools, pFIB-c and pFIB-6, have been developed to facilitate the exclusion of significant liver fibrosis in children with MASLD.^45^ These scores incorporate variables including sex, ethnicity, weight z-score, homeostatic model assessment–insulin resistance, ALT levels, and hypertension status. Both models demonstrated strong discrimination in derivation and elastography-based validation cohorts, with c-statistics of 0.839 (pFIB-c) and 0.826 (pFIB-6), and negative predictive values exceeding 90%.^45^ However, their performance declined in histologically confirmed and tertiary care cohorts, settings characterized by higher prevalence of advanced fibrosis and elevated ALT, where AUCs ranged from 0.710 to 0.770.^45^

Taken together, current evidence supports a tiered approach to pediatric MASLD screening: starting with accessible tools such as ALT and WHtR in primary care and school-based settings, followed by the application of non-invasive fibrosis scores for stratifying risk in confirmed cases. Ongoing validation and refinement of these models will be essential for establishing standardized screening algorithms that balance feasibility, accuracy, and cost-effectiveness across diverse pediatric populations.

## Diagnosis of pediatric MASLD

The diagnostic criteria for MASLD were updated in 2023, with a particular emphasis on the coexistence of hepatic steatosis and at least one of five cardiometabolic risk factors.^3^ Given that hepatic steatosis is a prerequisite for diagnosis, its accurate identification is critical. There are many tools for this purpose, among which magnetic resonance imaging–proton density fat fraction (MRI-PDFF) is currently the most accurate and validated tool for quantifying hepatic steatosis in children. When acquired using multi-echo Dixon techniques, MRI-PDFF demonstrates excellent correlation with magnetic resonance spectroscopy and meets the technical standards established by the Quantitative Imaging Biomarkers Alliance.^3^ Its reproducibility and precision have been confirmed in multiple pediatric populations, including those in China.^46^ A meta-analysis involving 874 children and adolescents with MASLD found that MRI-PDFF accurately diagnosed S1-3 steatosis, with a summary sensitivity of 95% and specificity of 92%.^47^ In contrast, abdominal ultrasound, despite its widespread use and accessibility, suffers from substantial operator dependence and subjective interpretation of liver echogenicity, which can be affected by coexisting obesity, inflammation, or fibrosis.^3^^,^^19^ Research from China has reported high sensitivity (91%) but poor specificity (57%) for ultrasound in detecting steatosis, indicating that while it may be suitable for initial screening, it lacks the diagnostic accuracy required for confirmation.^48^^,^^49^

Controlled attenuation parameter (CAP), measured via vibration-controlled transient elastography, provides a semi-quantitative estimate of liver fat by assessing ultrasound signal attenuation.^3^ CAP has shown moderate diagnostic utility for detecting moderate-to-severe steatosis in obese pediatric populations, particularly in research settings.^19^^,^^50^ A meta-analysis shows that CAP accurately diagnosed S1-3 steatosis, with a summary sensitivity of 0.86 (95% CI, 0.70–0.94) and specificity of 0.88 (95% CI, 0.71–0.96).^47^ However, pediatric-specific diagnostic thresholds remain undefined, and the current evidence does not support their routine clinical use.^3^ As such, while imaging-based techniques continue to evolve, their applicability in everyday clinical practice still depends on further validation and standardization.

Effective non-invasive diagnostic tools in pediatric populations remain limited. A study validated the predictive performance of the fatty liver index (FLI) and hepatic steatosis index (HSI) in diagnosing MASLD in the young population.^51^ The AUCs of FLI and HSI for MASLD were 0.91 and 0.90 in the NHANES data, respectively, and 0.93 for both indices in real-world clinical data. The optimal cut-offs for detecting hepatic steatosis were 20 (lower) and 50 (upper) for the FLI and 30 (lower) and 40 (upper) for the HSI. For severe MASLD, the FLI cut-off increased to 60, while the HSI cut-off remained at 40, with both achieving AUCs of 0.83.^51^ The cut-offs were mainly derived from the Korean population and still need to be evaluated for their effectiveness in Chinese children.

In terms of blood-based biomarkers, no serum test has been widely validated for the noninvasive diagnosis of hepatic steatosis in children. Prior research investigating markers such as adiponectin and fibroblast growth factor-21 has been limited by small sample sizes and lack of imaging or histological confirmation.^52^ More recently, integrated diagnostic models based on anthropometric and routine laboratory data have shown promise. A study conducted in China reported that a liver fat content prediction model demonstrated strong performance, achieving an AUC of 0.90 in both internal and external validation cohorts, and sensitivity and specificity ranging from 0.82 to 0.92 and 0.82 to 0.90, respectively.^53^ While these tools offer potential for early risk stratification, they remain investigational and require external validation before broader clinical adoption.

Once steatosis and metabolic dysfunction are identified, alternative causes of liver disease should be systematically excluded. These include drug-induced liver injury, viral hepatitis, autoimmune hepatitis, Wilson disease, celiac disease, and, in rare cases, monogenic metabolic disorders.^3^ In children of persistently elevated liver enzymes or diagnostic uncertainty, liver biopsy remains the gold standard, though its invasive nature necessitates judicious use.^54^

Taken together, the current diagnostic framework for pediatric MASLD integrates ALT screening, imaging confirmation, and selective use of biopsy. Although MRI-PDFF remains the most accurate modality, its availability is limited. Ultrasound and CAP offer practical but imperfect alternatives, while serum-based models hold promise for future use pending validation. Further refinement and integration of these modalities are essential to support early and scalable diagnosis in pediatric populations.

## Clinical management of pediatric MASLD

Lifestyle modification remains the first-line and most effective approach for the treatment and management of pediatric MASLD.^3^ Numerous clinical studies have shown that combined diet and exercise interventions can significantly reduce hepatic steatosis, as assessed by imaging modalities such as MRI, and are accompanied by reductions in body mass index (BMI) or overall body weight.^55^

For children with MASLD, it is recommended to control the total energy intake, limit the consumption of free sugars and saturated fats. In a randomized controlled trial (RCT) focused on adolescent boys with MASLD, an 8 weeks of provision of a diet low in free sugar content significantly improved hepatic steatosis compared with the usual diet.^56^ Research also indicates that both a low-carbohydrate diet and a low-fat diet can effectively reduce liver fat content, lower BMI, and decrease ALT levels in obese children with MASLD over a period of six months.^57^

Evidence from China highlights the importance of individual behaviors, such as healthy diet and physical activity, as well as environmental factors, including family and school influences. A study involving 1392 children with obesity implemented a comprehensive lifestyle program. After follow-up, the intervention group exhibited a significant reduction in BMI compared to controls (ΔBMI: −0.46; 95% CI: −0.67 to −0.25; p < 0.001) and a 27.0% relative decrease in obesity prevalence, compared to just 5.6% in the control group.^58^ Importantly, with MASLD now documented in preschool-aged children, early interventions are gaining relevance. A study in China enrolled children aged 3–6 years across six kindergartens. After 12 months, the intervention group had a smaller increase in BMI z-score (0.24 vs. 0.41), with an adjusted between-group difference of −0.31 (95% CI: −0.47 to −0.15), and a significantly lower risk of overweight/obesity (OR: 0.43; 95% CI: 0.19–0.96).^59^

These findings highlight the importance of early, family-centered interventions, despite practical challenges like competing family priorities, intergenerational dynamics in childcare, and socioeconomic constraints. School-based lifestyle programs are also crucial for preventing and managing MASLD in children. However, the aforementioned studies mainly focus on obesity rather than MASLD. Future research should pay more attention to high-quality RCT studies based on Chinese data to provide evidence for interventions that reduce BMI in children with MASLD.

The 24-Hour Movement Guidelines (24-HMG) offer a holistic framework for optimizing children's daily behaviors by integrating recommendations for physical activity, sleep, and screen time. They advocate for at least 60 min of moderate-to-vigorous physical activity per day, age-appropriate sleep durations (9–11 h for school-age children; 8–10 h for adolescents), and limited recreational screen time to no more than 2 h per day.^60^ Cross-sectional studies indicate that meeting at least two of the 24-HMG components significantly reduces the odds of general obesity.^61^ Furthermore, greater adherence to the 24-HMG recommendations was associated with lower continuous metabolic syndrome score in Chinese children and adolescents.^62^ These results may support the integration of 24-HMG into public health campaigns and school curricula as part of MASLD prevention strategies in pediatric populations. However, high-quality clinical evidence is still needed to confirm the specific benefits of 24-HMG for pediatric MASLD.

Currently, pharmacological treatment options for pediatric MASLD are extremely limited and should be considered only as adjuncts to lifestyle modification.^61^^,^^63^ No medications are specifically approved for MASLD in children, and available agents provide only modest benefits in selected populations. One of the most promising classes of drugs for pediatric MASLD is glucagon-like peptide-1 receptor agonists (GLP-1 RAs), which have shown significant efficacy in treating obesity and related metabolic disorders. A recent RCT showed that among adolescents with obesity, a once-weekly 2.4-mg dose of semaglutide, a GLP-1 RAs, combined with lifestyle intervention leads to a greater reduction in BMI, decreasing by 16.7% from baseline, compared to lifestyle intervention alone.^64^ Additionally, improvements in cardiometabolic risk factors were more significant.^64^ According to the Chinese MASLD pediatric consensus recommendations, for children ≥12 years with MASLD, severe obesity and/or type 2 diabetes, and who have not improved after 6 months of lifestyle interventions, the cautious use of GLP-1 RAs is recommended.^65^ Metformin, a widely used drug for managing type 2 diabetes, has also been explored as a treatment option for pediatric MAFLD. A meta-analysis showed that while metformin does not significantly improve liver enzymes, it may offer some benefits in improving lipid profiles and insulin metabolism in pediatric MAFLD.^66^ Current guidelines do not recommend metformin as a first-line therapy for children with MASLD; however, it may be considered in patients aged ≥10 years with insulin resistance.^65^^,^^66^ Statins have shown potential benefits in adult MASLD by slowing fibrosis progression and reducing the risk of liver-related events.^67^ However, their safety and efficacy in children remain to be established through further research. For pediatric patients with MASLD without hypercholesterolemia, statins are not recommended. In children aged 8 and above with MASLD and hypercholesterolemia, statins may be considered if low-density lipoprotein cholesterol remains ≥4.1 mmol/L despite 6 months of lifestyle modification; not recommended for those without hypercholesterolemia.^65^ Vitamin E, an antioxidant, has demonstrated some efficacy in improving liver enzymes and BMI in pediatric MASLD patients.^68^ However, given the limited evidence and potential long-term risks, vitamin E is not recommended as first-line therapy for pediatric MASLD and should be reserved for children with histologically confirmed MASH (Supplementary Table 1).^65^

In summary, lifestyle modification remains the cornerstone of treatment for pediatric MASLD, with recommendations to control the total energy intake and limit the consumption of free sugars and saturated fats. Pharmacological treatments for pediatric MASLD are very limited, provide only modest benefits in select cases, and should be reserved for children who do not respond adequately to lifestyle changes or who present with more advanced disease. Most evidence for pharmacological treatments comes from studies conducted in populations outside China, highlighting the urgent need for well-designed clinical trials in Chinese children to evaluate safety and efficacy and to inform population-specific treatment guidelines.

## Public health

The rising prevalence of childhood and adolescent overweight and obesity, which are central contributors to MASLD, not only threatens individual health outcomes but also imposes substantial economic costs, posing a significant barrier to achieving global public health goals.^69^ In recognition of this growing crisis, a Sino-international expert panel convened to develop a consensus statement aimed at advancing China's “Healthy China 2030” policy agenda.^70^ This consensus emphasized the central role of moderate-to-vigorous physical activity in improving metabolic and overall health among children and adolescents and advocated for comprehensive, population-level strategies to address childhood obesity and its related complications. Accordingly, the consensus provides pragmatic guidance for under-resourced areas, including school-based screening protocols and tailored approaches for ethnic minorities, directly supporting Healthy China 2030's equity goals.

To translate these recommendations into actionable policies, economic modeling has been employed to guide the design and prioritization of large-scale interventions. Investment case analyses have projected the health and economic impacts of childhood and adolescent overweight and obesity in children and adolescents aged 0–19, starting in 2025, accounting for healthcare costs, years of life lost, reduced wages, and productivity losses. Status quo projections estimate lifetime costs of $33 trillion in China, whereas implementation of priority interventions-including fiscal policies, social marketing, breastfeeding promotion, school-based policies, and nutritional counseling-could reduce costs by $2 trillion, yielding a return on investment (ROI) of $75 per $1 invested.^69^ A subsequent deterministic Markov cohort model projected that without intervention, China would experience 3.3 billion disability-adjusted life years (DALYs) and a lifetime economic impact of CNY 218 trillion (USD 31.6 trillion), equivalent to CNY 2.5 million (USD 350 thousand) per affected child. Implementation of five key interventions nationwide could avert 179.4 million DALYs and generate CNY 13.1 trillion in benefits over the cohort's lifetime.^71^

Given that obesity is a major driver of MASLD, the substantial economic and health returns from obesity interventions suggest that similar investments in MASLD prevention, particularly through early-life lifestyle interventions, could yield benefits by mitigating a cascade of downstream metabolic complications. However, this still requires forward-looking analysis and validation. Two more issues need to be considered when formulating policies. First, although the overlap between MASLD and alcohol-associated liver injury has been recognized, it has not been adequately considered in the context of underappreciated alcohol use in adolescence.^3^ A study surveyed 33,427 Chinese adolescents. 59.2% of the adolescents had consumed alcohol, with 29.6% adolescents starting to drink between the ages of 8 and 15.^72^ This high prevalence of early alcohol initiation, combined with the rising burden of MASLD, underscores the urgent need for government action. Second, Processed foods high in refined carbohydrates, such as instant noodles, bread, and sugary drinks, contribute to obesity and insulin resistance, driving MASLD despite adequate calories.^3^^,^^73^ Furthermore, food insecurity, linked to social disadvantages like low income and poor access to healthcare, exacerbates this double burden of malnutrition and overnutrition.

Despite the mounting evidence supporting the need for action, China's overall policy preparedness for MASLD remains at a relatively low level. Among the 12 countries in the East Asia and Pacific region, including China, none had developed or implemented a national or sub-national strategy specifically for MASLD, nor was MASLD mentioned in strategic documents for related conditions such as obesity, diabetes, or liver diseases. Although China is one of the few countries among the 32 with national MASLD clinical guidelines, its preparedness in key areas such as policy development, public awareness raising, epidemiological data collection, MASLD screening, and integrated care management is still insufficient, and an overall response system has yet to be established. This reflects that current attention and systematic response to MASLD as a public health challenge in China still need strengthening. This gap in systemic response is particularly concerning for the pediatric population, among whom the prevalence of MASLD is rising alongside childhood obesity, yet remains largely unaddressed in current national health strategies.^74^

## Future

Looking ahead, closing the identified gaps will require a nationally coordinated effort focused on generating robust Chinese-specific evidence and building effective, equitable systems for prevention and care. Future work must prioritize the establishment of a comprehensive, nationally representative epidemiological surveillance system to accurately monitor the prevalence and progression of pediatric MASLD across diverse regions and demographics. There is an urgent need to develop and validate cost-effective, standardized screening algorithms that are feasible for implementation in primary care and school settings, particularly in underserved rural areas. Well-designed, longitudinal RCTs are critically needed to evaluate the efficacy of tailored lifestyle interventions and to explore safe, effective pharmacological treatments specifically for Chinese children with MASLD. Simultaneously, clinical pathways must be clearly defined to integrate screening, diagnosis, and multidisciplinary management across different levels of the healthcare system. Ultimately, these efforts should be underpinned by stronger policy commitment, including the formulation of a national MASLD strategy, and explicitly address the unique vulnerabilities of the pediatric population.

The Liver Health Consortium in China (CHESS) and the National Center for Children's Health have established the China Children's Liver Health Collaborative Group. This collaborative initiative is actively building multi-center, large-scale databases and biobanks to map pediatric MASLD and liver fibrosis prevalence, conducting nationwide surveys to elucidate risk factors and inform policy, developing standardized non-invasive liver fibrosis models accessible without specific ultrasound equipment, performing lipoprotein subfraction testing for improved risk stratification, and creating predictive models for pediatric MASLD risk, investigating the efficacy of exercise interventions through randomized controlled trials. These concerted efforts aim to contribute significantly to safeguarding the liver health of Chinese children.

## Conclusion

Pediatric MASLD in China is an emerging public health concern, driven by the intersection of shifting lifestyles, early-onset metabolic dysfunction, and widening health disparities. While prevalence remains moderate, its upward trend, regional clustering, and onset in early childhood signal a critical juncture for intervention. Current evidence underscores the value of early, scalable screening and lifestyle-based management, yet gaps persist in standardized diagnostic pathways, fibrosis risk stratification, and equitable policy implementation. Moving forward, a nationally coordinated, life-course approach, integrating clinical care, public health infrastructure, and socioeconomic context, is essential to curbing the long-term burden of MASLD and its associated comorbidities in the next generation.

## Contributors

XGL: Conceptualisation, Formal analysis, Investigation, Methodology, Visualisation, Writing–review & editing; XDZ: Conceptualisation, Data curation, Formal analysis, Investigation, Methodology, Visualisation, Writing–review & editing; JW: Conceptualisation, Investigation, Methodology, Visualisation, Writing–review & editing; ZZ: Conceptualization, Investigation, Methodology, Validation, Writing–review & editing; FX: Investigation, Methodology, Validation, Visualisation; YLL: Investigation, Methodology, Validation, Visualization; WHL: Investigation, Methodology; XSY: Methodology, Validation; SMS: Conceptualisation, Visualisation; LTZ: Methodology, Data interpretation, Project administration; MHZ: Methodology, Data interpretation; YMN: Conceptualisation, Methodology, Resources, Writing–review & editing; Supervision; XLQ: Conceptualisation, Data curation, Investigation, Methodology, Project administration, Resources, Funding, Supervision, Writing–review & editing.

## Declaration of interests

There are no conflicts of interest associated with this paper.
