# Supplementary material for: Pediatric MASLD in China: epidemiology, screening, diagnosis, and management
Source: Lancet Reg Health West Pac. 2025 Oct 18;64:101717. doi: 10.1016/j.lanwpc.2025.101717 (PMC12556317; doi:10.1016/j.lanwpc.2025.101717)
Supplement: Supplementary Table 1 [file mmc1.docx]

**Supplementary Table 1.** Pharmacological management strategies for pediatric MASLD: 2025 expert consensus recommendations.

| **Intervention** | **Recommendations** |
| --- | --- |
| **GLP-1 RAs** | Children ≥12 years with MASLD, severe obesity and/or type 2 diabetes, and who have not improved after 6 months of lifestyle interventions; cautious use is recommended. |
| **Metformin** | Children ≥10 years with insulin resistance; not recommended as a first-line therapy, but may be considered in selected patients. |
| **Statins** | Children ≥8 years with MASLD and hypercholesterolemia (LDL‑C ≥4.1 mmol/L) despite 6 months of lifestyle modification; may be considered; not recommended for those without hypercholesterolemia |
| **Vitamin E** | Children with histologically confirmed MASH; not recommended as first-line therapy. |

GLP-1 RAs=glucagon-like peptide-1 receptor agonists; MASLD = metabolic dysfunction-associated steatotic liver disease; MASH= metabolic dysfunction-associated steatohepatitis.
