# Supplementary figures and images for: Pediatric MASLD in China: epidemiology, screening, diagnosis, and management
Source: Lancet Reg Health West Pac. 2025 Oct 18;64:101717. doi: 10.1016/j.lanwpc.2025.101717 (PMC12556317; doi:10.1016/j.lanwpc.2025.101717)

**Distribution of region among general and obese children with MASLD**

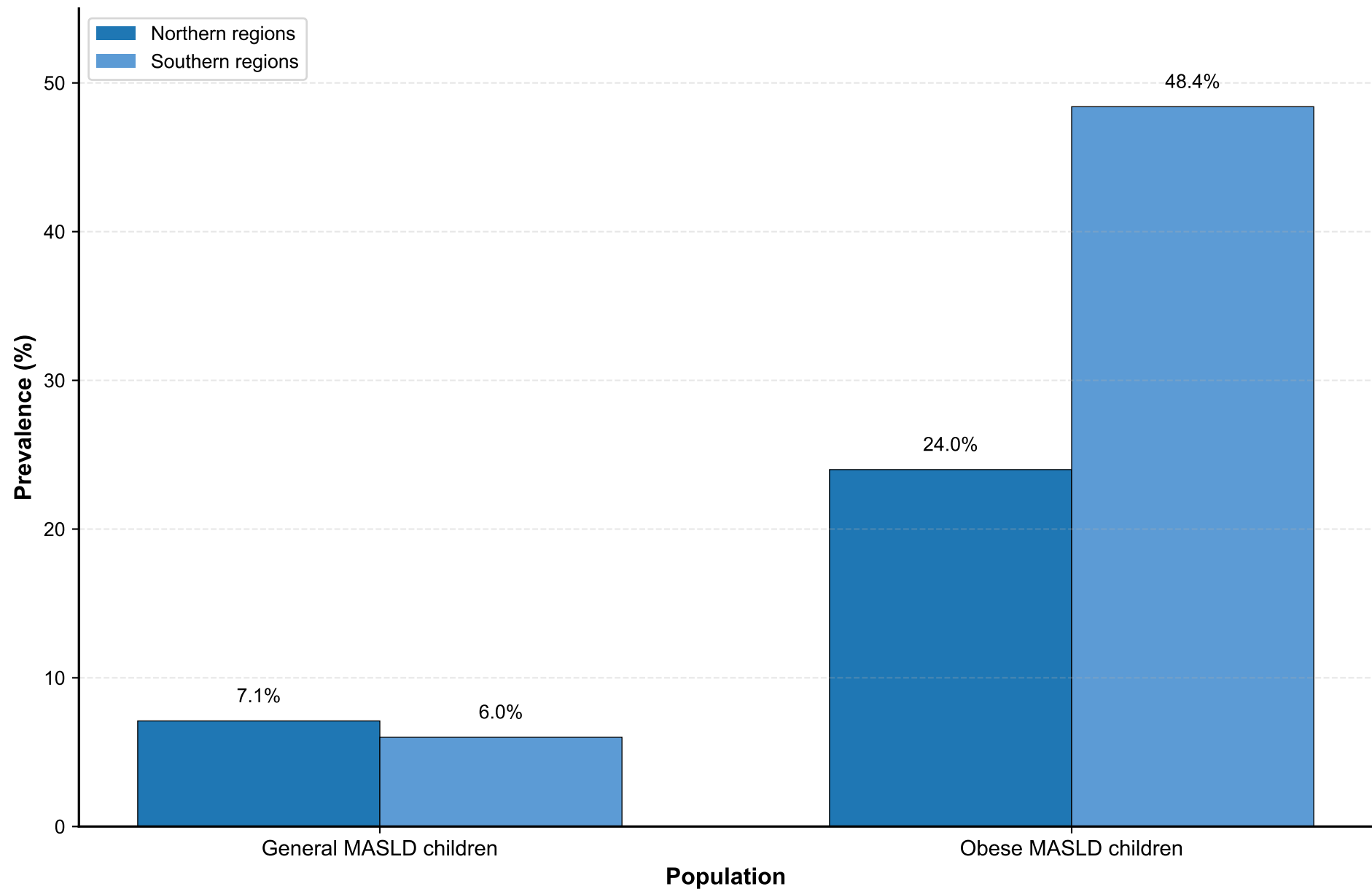

Supplement: Supplementary Figure 1 [file mmc2.pdf]
