# Supplementary material for: Pediatric MASLD in China: epidemiology, screening, diagnosis, and management
Source: Lancet Reg Health West Pac. 2025 Oct 18;64:101717. doi: 10.1016/j.lanwpc.2025.101717 (PMC12556317; doi:10.1016/j.lanwpc.2025.101717)
Supplement: Supplementary Data [file mmc3.docx]

Key findings from the return on investment for the prevention and treatment of childhood and adolescent overweight and obesity in China

| **Parameter** | **Status Quo Scenario (No Intervention)** | **Intervention Scenario (All Five Interventions)** |
| --- | --- | --- |
| **Study Design** | Deterministic Markov cohort model | Deterministic Markov cohort model |
| **Time Horizon** | 2025–2092 | 2025–2092 |
| **Target Population** | Children and adolescents aged 0–19 in 2025 | Children and adolescents aged 0–19 in 2025 |
| **Health Burden** | 3.3 billion disability-adjusted life years (DALYs) | 179.4 million DALYs averted |
| **Economic Impact** | CNY 218 trillion (USD 31.6 trillion) lifetime economic impact | CNY 13.1 trillion lifetime benefits |
| **Per Capita Economic Impact** | CNY 2.5 million (USD 350,000) per affected child/adolescent | CNY 177,745 benefits per child/adolescent nationally |
| **Healthcare Costs** | CNY 261 billion direct healthcare costs | CNY 71.2 billion healthcare expenditure reduction |
| **Productivity Loss** | CNY 4.6 trillion productivity loss | CNY 637.6 billion production losses averted |
| **Wage Impacts** | CNY 1.8 trillion lost wages | CNY 934.3 billion wage gains |
| **Value of Life Lost** | CNY 211.4 trillion | Not specified in intervention scenario |
| **Gender Disparity** | 75% of DALYs occur among girls | Not specified |
| **Annual Economic Burden** | Equivalent to 3% of China's annual GDP | Not specified |
| **Implementation Cost** | Not applicable | CNY 146 billion total implementation cost (2025–2044) |
| **Return on Investment** | Not applicable | 89:1 ROI over lifetime (95% CI: 4 to 141) |

**Interventions Included:** 20% excise tax on sugar-sweetened beverages, e-Health breastfeeding promotion, restrictions on marketing unhealthy foods to children, combined school-based interventions, nutrition counselling by physicians.
